# A New Efficient Method for Detecting Phase Singularity in Cardiac Fibrillation

**DOI:** 10.1371/journal.pone.0167567

**Published:** 2016-12-01

**Authors:** Young-Seon Lee, Jun-Seop Song, Minki Hwang, Byounghyun Lim, Boyoung Joung, Hui-Nam Pak

**Affiliations:** Yonsei University Health System, Seoul, Korea; Indiana University, UNITED STATES

## Abstract

**Background:**

The point of phase singularity (PS) is considered to represent a spiral wave core or a rotor in cardiac fibrillation. Computational efficiency is important for detection of PS in clinical electrophysiology. We developed a novel algorithm for highly efficient and robust detection of PS.

**Methods:**

In contrast to the conventional method, which calculates PS based on the line integral of the phase around a PS point equal to ±2π (the Iyer-Gray method), the proposed algorithm (the location-centric method) looks for the phase discontinuity point at which PS actually occurs. We tested the efficiency and robustness of these two methods in a two-dimensional mathematical model of atrial fibrillation (AF), with and without remodeling of ionic currents.

**Results:**

1. There was a significant association, in terms of the Hausdorff distance (3.30 ± 0.0 mm), between the PS points measured using the Iyer-Gray and location-centric methods, with almost identical PS trajectories generated by the two methods. 2. For the condition of electrical remodeling of AF (0.3 × I_CaL_), the PS points calculated by the two methods were satisfactorily co-localized (with the Hausdorff distance of 1.64 ± 0.09 mm). 3. The proposed location-centric method was substantially more efficient than the Iyer-Gray method, with a 28.6-fold and 28.2-fold shorter run times for the control and remodeling scenarios, respectively.

**Conclusion:**

We propose a new location-centric method for calculating PS, which is robust and more efficient compared with the conventionally used method.

## Introduction

Ventricular fibrillation (VF) is the most common cause of death for patients with structural heart disease [1, 2], and atrial fibrillation (AF) is associated with 15~25% cause of ischemic stroke [3]. However, mechanisms of cardiac fibrillation remain elusive. The multiple wavelet hypothesis [4] predicates that a continuous wave-break maintains fibrillation by collisions and break-up of wavelets and creation of new rotors. In addition, the focal source hypothesis explains the maintenance of fibrillation by stable periodic sources and centrifugal fibrillatory conduction [5]. Phase singularity (PS) point, which corresponds to a wave-break point [6] or a fibrillatory rotor [7], has been defined as a point that does not have a definite phase while its neighboring sites exhibit phases that change continuously from –π to +π [2]. Pak et al. [8] reported ablation of PS points located at papillary muscle terminated VF, and Narayan et al. [9] observed rotors in AF patients, with rotor ablation effectively terminating AF. However, detecting PS points from fibrillatory spiral waves is a complicated procedure, and thus requires to define a descriptor for time- and space-dependent progression of action potentials, which is defined as a phase [10]. Since earlier studies tracked the spiral wave tip using the concept of isopotential [11, 12], several automatic methods for PS detection have been developed [13, 14]. Iyer and Gray developed an automatic method for calculating PS using the line integration around the PS point [14]. However, one challenging problem is that calculation of PS points is time-consuming when using conventional algorithms in high-dimensional tissue models [15]. Here, we have developed a new efficient location-centric method for identifying PS points. Our new method for detecting PS depends only on the change in voltage at a local site. This feature shows a clear contrast with the conventional method by Iyer-Gray, which requires voltage information at the neighboring sites. Thus, we have called this new method 'local-centric' to represent a method relying on a local value of voltage.

We demonstrate that the proposed location-centric method for calculating PS points is more efficient than the classical Iyer-Gray method, while at the same time yielding almost the same accuracy for a two-dimensional (2D) human atrial tissue model.

## Methods

We have developed a new method for identifying PS of spiral waves observed in atrial fibrillation. We tested the method’s performance on two different types of spiral waves: an unstable one (control case) and a stable one (0.3 × I_CaL_). Numerical simulations using a 2D tissue model of human atrial cells were performed for two different model scenarios: a control setting with original parameters, and a setting in which the current due to the model L-type Ca^2+^ channels was reduced by 70% alone (0.3 × I_CaL_).

### Simulation of spiral waves

Action potentials in atrial cells in AF were modeled following Courtemanche et al. [16], and spatiotemporal wave propagation in a 2D tissue was modeled by the following equation [17]:

$$\frac{\partial \text{V}}{\partial \text{t}}\text{=}\partial {\text{-(I}}_{\text{ion}}{\text{+I}}_{\text{stim}}{\text{)/C}}_{\text{m}}\text{+D(}\frac{\partial^{\text{2}}\text{V}}{\partial {\text{x}}^{\text{2}}}\text{+}\frac{\partial^{\text{2}}\text{V}}{\partial {\text{y}}^{\text{2}}})$$

where I_ion_ denotes the sum of all ionic currents, I_stim_ is a stimulating current, and V denotes the transmembrane potential. The parameter D = 0.001 cm^2^/ms is the diffusion coefficient [18] and C_m_ = 1 μF/cm^2^ is the membrane capacitance. The model 2D tissue had the dimensions of 15 cm × 15 cm, with each node representing one cell. The time step was adaptively varied between 0.01 and 0.1 ms, and the data sampling interval was 1 ms [19]. The standard cross-field protocol was used for initiating a spiral wave; the protocol consisted of applying a vertical field stimulation (S1) followed by a horizontal field stimulation (S2), with a coupling interval of 300 ms.

### Methods for PS calculation

In 2001, Iyer and Gray presented a method for accurate localization of PS [14]. In fact, their method was based on the previously developed method by Gray [2]. The phase (θ) at each site (x,y) was calculated as

$$\theta \left(x,y,t\right)=\text{arctan}\left[V\left(t+\tau \right)-V_{mean},V\left(t\right)-V_{mean}\right]$$

where the function arctan calculated the phase difference between the membrane potential at time t (V(t)) and the delayed transmembrane potential at time t+τ (V(t+τ)), with the time delay τ = 30 ms. Gray et al. used a τ value of τ = 25 ms in their original paper [2] for fluorescence measurement from a site.

At each site (x,y), we calculated V_mean_(x,y) by averaging the action potential during the whole fibrillation state [15]. V_mean_ is an important value, that plays a role as the origin point in the phase space V(t) and V(t+τ). Gray et al. used the value of fluorescence signals (F_mean_) of transmembrane from a site as a center of the phase space.

In this method, the PS is identified when the following condition is satisfied:

∮∇θ⋅dr=±2π.

**Fig 1:**
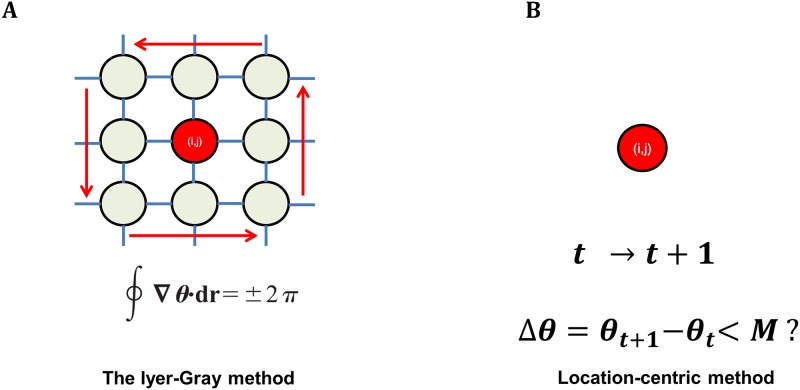
Schematics comparison of the Iyer-Gray and location-centric method. A. The Iyer-Gray method calculates phases that range from –π to π and calculates the line integral of the phases at a candidate point with its eight neighbor points. B. The location-centric method searches for a singularity point at a candidate point, subject only to one condition, θ_n+1_—θ_n_ < M.

Thus, the line integral of the phase change, along a small-radius path surrounding the site becomes ±2π. In other words, using the Iyer-Gray method we need to calculate the line integral at a probing point with its eight neighbor points. Fig 1A shows the flowchart describing the steps of PS calculation using the Iyer-Gray method: eight neighbor sites around a candidate site denoted by an index (i,j) are needed for computing the line integral, and at each site the phase is determined by conditions that ensure the phase is in the –π to π range.

In this study, we propose a novel method for PS calculation. We suggest to call this method ‘location-centric’, because the PS point is selected only based on the phase difference (Δθ) at a local site, without checking for phase changes at the site’s neighbor points (Fig 1B). Thus, a candidate site is selected as the PS point if the phase difference (Δθ) at the site is below a threshold value:

$$\Delta \theta =\theta_{n+1}-\theta_{n}<M$$

where θ_n_ is the phase in the nth time frame and M is set to -π. The pseudocode of the algorithm is described in the S1 Appendix.

### Calculation of similarity metric between two methods

To compare the extent of similarity between the PS trajectories generated by the two methods, we chose the Hausdorff distance [20]. This measure captures the similarity between two sets of PS trajectories by calculating the maximal discrepancy between them. Let A = {*p*_1_, *p*_2_, …, *p*_*n*_} and B = {*q*_1_, *q*_2_, …, *q*_*m*_} be two sets of PS points obtained by the Iyer-Gray and the location-centric methods, respectively. The Hausdorff distance is defined as

H(A,B)=max(h(A,B),h(B,A))

where h(A, B) = *max*_*p*∈*A*_ *min*_*q*∈*B*_ ∥*p* − *q*∥ and ∥·∥ denotes the Euclidean norm.

We also compared computation times for calculating PS points by the two different methods in the control scenario and scenario of electrical remodeling (0.3 × I_CaL_), respectively. We measured the total computation time required by the Iyer-Gray method and the proposed location-centric method, for the calculation and detection of the same PS points, in the same condition (1 s of simulation time); for this purpose, we used the ‘tic and toc’ commands in MATLAB (MathWorks, Inc.), excluding the data transfer time.

The spatial step size for computation of the model was 0.25 mm. However, when we illustrated the 2D images in the figure, the actual spatial resolution was 0.5 mm. Thus, two adjacent pixels in the result images are 0.5 mm apart. We calculated the Hausdorff distance based on the actual spatial resolution.

## Results

### Comparison of the location-centric and the Iyer-Gray methods

**Fig 2:**
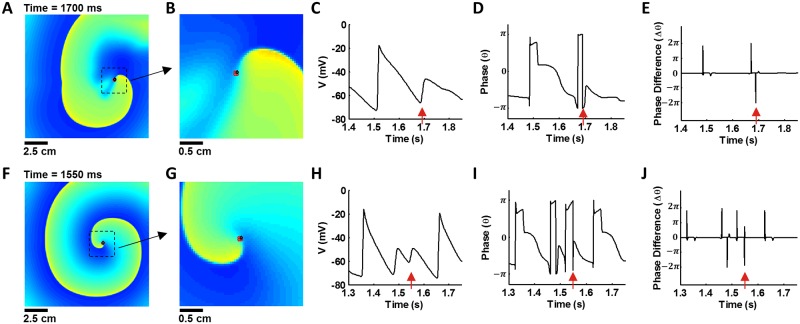
PS detection using the two methods. PS points calculated using the Iyer-Gray method (red circles) and the location-centric method (black diamonds). Snapshots of model dynamics, showing an unstable (control, panels A–E) and a stable (0.3 × I_CaL_, panels F–J) spiral wave. The PS points calculated by the two methods overlapped in the 2D map. B, G: zoomed images. C, H: Action potentials at PS points. D, I: Phases. E, J: Phase differences.

We compared the PS locations determined by the Iyer-Gray method (red circles in Fig 2) and by the location-centric method (open black diamonds in Fig 2), for the control condition (Fig 2A–2E) and the electrical remodeling condition (0.3 × I_CaL_, Fig 2F–2J), respectively. For plotting the PS points determined by the location-centric method, we calculated PS points with the temporal resolution of 1 ms (Fig 2B and 2G) and sampled the PS points at the phase difference (Δθ) below the threshold level of -π (M, Fig 2E and 2J). The PS points calculated at 1,550 ms by both methods almost overlapped on the 2D maps, for the control and electrical remodeling scenarios. The phases (θ) are calculated every 1 ms (Fig 2). At a site without a singularity all phases change continuously, except during the activation time of an action potential. During this period, the phase difference (Δθ) is positive. However, if a site experiences a PS, the phase difference (Δθ) decreases abruptly and yields a large negative value (Fig 2E and 2J). We set the threshold of Δθ to –π, and the spatiotemporal locations of the values below the threshold level (M) were marked on the 2D voltage maps as the PS points calculated by the ‘location-centric method’ (Fig 2A and 2F, open black diamonds). Thus, PS detection condition (Δθ = θ_n+1_—θ_n_ < M) shows a clear distinction of the proposed method from the Iyer-Gray method, because the latter requires integrating around all sites for determining a PS point. We added two zoomed panels (Fig 2B and 2G) to see detailed images of identified PSs, which are shown as a cluster of four PS points (red colored circles) that were obtained by the Iyer-Gray method and one PS point (open black diamond). Fig 2G displayed that an open black diamond, calculated by location-centric method, overlapped with one PS in the cluster of four PS points obtained by the Iyer-Gray method. To show examples where PS points detected by the location-centric method are different from those of the Iyer-Gray method, we added snapshot images of the two cases (control and 0.3×I_CaL_ cases) near the spiral wave core and their corresponding membrane potentials (S1 Fig).

**Fig 3:**
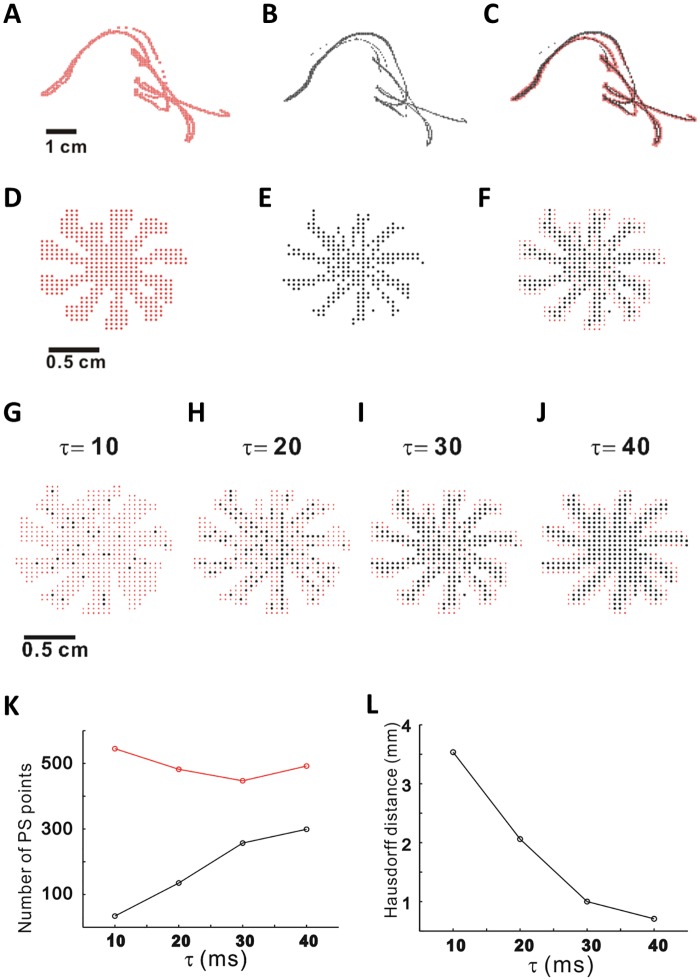
Spatial distributions of PS trajectories and effects of time delay on PS calculation. PS trajectories for the control scenario: (A) using Iyer-Gray method, (B) using location-centric method, (C) combined image. PS trajectories for the 0.3×I_CaL_ scenario: (D) using Iyer-Gray method, (E) using location-centric method, (F) combined image. (G-J) PS points were overlapped for different τ (time delay values) = 10 ms (G), 20 ms (H), 30 ms (I), and 40 ms (J). The number of PS points (K), and Hausdorff distance (L) of Iyer-Gray method (red dots) and location-centric method (black dots).

To compare the spatial distributions of the calculated PS points, the spatial trajectories of the PS points obtained by the two methods were identified and their traces were plotted in the 1,130–2,130 ms time interval (Fig 3). Because electrical remodeling of AF accompanies down-regulation of I_CaL_, which stabilizes localized reentry, we tested the Iyer-Gray method (red dots in Fig 3) and the location-centric method (black dots in Fig 3) performance on the PS detection in the control condition (Fig 3A–3C) and in the condition of reduced Ca^2+^ current (0.3 × I_CaL_, Fig 3D–3F). Consistent with the control condition, the PS trajectories calculated by the two methods for the condition of 0.3 × I_CaL_ matched satisfactorily, yielding stable spiral waves.

### Effects of model parameters on PS calculation

We explored the effect of time delay on PS calculation using two methods. For four different time delays (τ = 10 ms, 20 ms, 30 ms and 40 ms), we illustrated overlapped images of PS points (Fig 3G–3J) in the case of 0.3×I_CaL_ calculated by the Iyer-Gray method (red dots) and the location centric method (black dots).

To find the change in the number of calculated PS points, we plotted the number of PS points using the Iyer-Gray method (red dots) and the location-centric method (black dots; Fig 3K). In the Fig 3L, Hausdorff distances were calculated with different time delays. It should be noted that the spatial similarity between the two methods, estimated by small Hausdorff distances (HD), increases for τ = 30 ms (HD = 1 mm) and 40 ms (HD = 0.71 mm).

**Fig 4:**
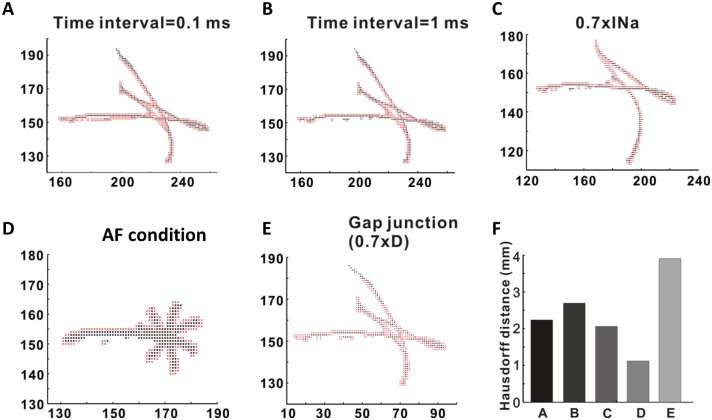
Parameter effects on PS calculation between the two methods. (A) and (B) show overlapped PS points found using the two methods in control condition (red: Iyer-Gray method; black: location-centric method) at two sampling time intervals (0.1 ms and 1 ms). We investigated the effects of electrical remodeling on PS detection: 0.7×I_Na_ (30% reduction in Na+ channel conductance) (C), AF condition, and 0.7×D (30% reduction in gap junctional coupling). For the AF condition, we applied the same condition that was used in our recent paper [19]: I_Na_ (−10%), I_to_ (−70%), I_CaL_ (−70%), I_Kur_ (−50%), SR Ca^2+^ leak (+25%), I_K1_ (+100%), I_NaCa(max)_ (+40%) (D). For the change in the gap junctional coupling caused by changing 0.3×D (E), we found that HD was 3.9 mm, which was the largest among five examples (F).

We performed an additional parameter sensitivity analyses to understand how the new method behaves as parameters change due to sampling time intervals of phase values and electrical remodeling. In Fig 4, panels A and B show overlapped PS points found using the two methods in the control condition (red: Iyer-Gray method; black: location-centric method) in two sampling time intervals of phase values 0.1 ms and 1 ms. We also investigated the effects of electrical remodeling on PS detection: 0.7×I_Na_ (30% reduction in Na^+^ channel conductance), AF condition, and 0.7×D (30% reduction in gap junctional coupling). For the AF condition, we applied the same condition that was used in our recent paper [19]: I_Na_ (−10%), I_to_ (−70%), I_CaL_ (−70%), I_Kur_ (−50%), SR Ca^2+^ leak (+25%), I_K1_ 1(+100%), and I_NaCa(max)_ (+40%). For two time spacings (0.1 ms and 1 ms), the Hausdorff distance showed a small difference between the two times: HD = 2.23 mm (T = 0.1 ms) and HD = 2.69 mm (T = 1 ms). The additional comparisons for T = 0.5 ms, 5 ms, and 10 ms were provided in S2 Fig. For the change in the gap junctional coupling caused by changing 0.3×D, we found that HD was 3.9 mm, which was the highest HD value among five examples.

### Robustness of location-centric PS for different threshold levels

**Fig 5:**
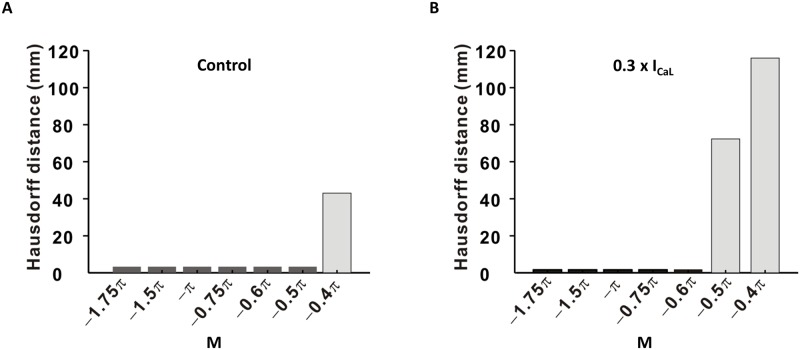
Quantitative comparison of spatial distributions in terms of the Hausdorff distance. Hausdorff distances between the results obtained by the location-centric method and the Iyer-Gray method were measured for different thresholds (M). (A) Hausdorff distances calculated for the control condition. (B) Hausdorff distances calculated for the 0.3 × I_CaL_ condition.

We tested the robustness of the two methods with respect to quantitative tracing of meandering trajectories of PS points during a 1-s-long AF recording. We calculated the Hausdorff distance between the PS points measured by the Iyer-Gray method and those measured by the location-centric method, for different phase difference thresholds of the location-centric method (Fig 5). The location-centric method consistently yielded small Hausdorff distances (3.30 ± 0.0 mm) for most of the threshold values M, except the values ≥ –0.4π (Fig 5). For the 0.3 × I_CaL_ condition, the Hausdorff distance was short (1.64 ± 0.09 mm) for the threshold M ≤ –0.6π for the location-centric method (Fig 5B). Therefore, for both conditions, the location-centric method generally exhibited consistently smaller values of the Hausdorff distance compared with the Iyer-Gray method.

### Efficiency of the location-centric method

**Fig 6:**
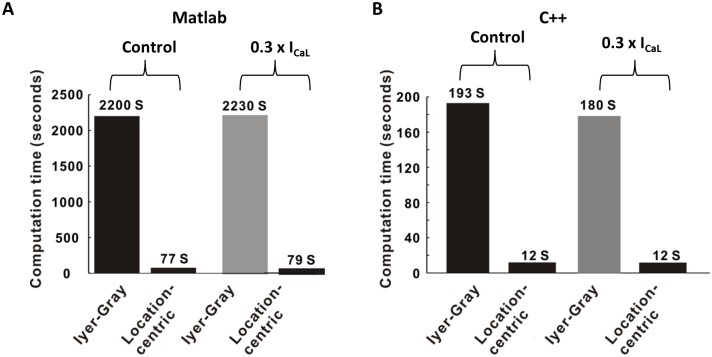
Comparison of computational times for PS calculations. PS calculation times when using the two methods for a 1-s simulation: for the control and remodeling (0.3×I_CaL_) scenarios. We tested programs with the Matlab package and C++.

Since the location-centric method for locating PS points of spiral waves uses the idea that PS points are points of discontinuity in the phase map, this method is more efficient for detecting PS points than the Iyer-Gray method. For the same condition, with a 1-s-long AF, the location-centric method consistently outperformed the Iyer-Gray method for both the control and electrical remodeling conditions (Fig 6). Therefore, the location-centric method exhibited a ~28-fold performance improvement over the Iyer-Gray method, for both conditions, which was measured by MATLAB simulation, excluding the data input and output time. We also implemented the same algorithms in C++ with the same data set. The results showed that there was a reduction in the factors of increase in speed: for the control (193 s vs. 12 s) and for 0.3×I_CaL_ (180 s vs. 12 s). Thus, there was ~16-fold increase in the speed of calculating PS when using the location-centric method.

## Discussion

### Main findings

In this study, we have suggested a novel location-centric method for calculating PS points, which is more efficient and robust compared with the conventional Iyer-Gray method.

We developed a new method (location-centric) while searching for a PS-detecting method in 3D atria. We observed that there is discontinuity in the phase when phase singularity exists. Thus, we empirically found a new method for detecting phase singularity without the calculation of a line integral. Mathematically, the targets of our new approach and the Iyer-Gray method are temporal singularity and spatial singularity of the phase function θ(x, y, t), respectively. We only considered negative jump of the phase (θ_n+1_-θ_n_<0), because phase 0 depolarization of action potential causes positive jump of the phase (θ_n+1_-θ_n_>0).

The location-centric method detects the point of phase discontinuity at which PS is located, instead of calculating a phase based on the line integral of the phase. The proposed method yielded results that were very similar to those obtained by the conventional method, as captured by the short Hausdorff distance, thus demonstrating the method’s accuracy. The proposed method’s robustness was demonstrated by comparing the results obtained by the two methods for both the control condition and the condition of electrical remodeling. The location-centric method required a substantially smaller computation time, and performed ~28-fold faster than the Iyer-Gray method.

### The importance of PS points in cardiac fibrillation

Cardiac fibrillation is well recognized as the main cause of sudden cardiac death or cardioembolic stroke [21, 22]. Fibrillatory state in the cardiac tissue corresponds to the state of abnormal electrical activity, involving spontaneous and irregular propagation of electrical waves, owing to which the heart loses its ability to contract synchronously [23]. Furthermore, VF or AF might be sustained owing to multiple wavelets [4], which are manifested as multiple fractionated waves; yet, single or several stable rotors can be sufficient for maintaining fibrillation [6]. A phase-space PS, in which spiral waves evolve around a PS point, can occur as a result of the breakage of a wave front [24, 25]. Winfree suggested that the source of fibrillation can be observed in the form of topological defects or PS in the phase map [2, 26]. Thus, tracking of PS points, which leads to rotors, may provide insights on the mechanisms of fibrillation. Gray et al. [2] observed PS on the ventricular surface of the isolated rabbit heart, and they introduced a novel analysis algorithm for reducing the data size to describe spatiotemporal patterns of fibrillation by its phase θ(x,y,t) around PS. They successfully demonstrated the formation and termination of rotors by identifying and analyzing PS points.

### Potential clinical applications of PS calculation

In a clinical setting, detection of PS points in patients with AF/VF can facilitate our understanding of the role of PS points in fibrillation [25]. For instance, a stable mother rotor is known to induce fibrillatory conduction, and thereby is a key for sustaining AF [5, 7, 27]. Haissaguerre et al. [28] suggested that organized and discrete sources of AF exist in human AF patients, and showed that a local radiofrequency ablation could terminate AF. Recently, Narayan et al. also observed electrical rotors in human AF patients using computational maps, and demonstrated successful termination of fibrillation using 64-pole basket catheters. Targeting and ablation of sources by Focal Impulse and Rotors Modulation (FIRM) ablation has been suggested as an effective and novel therapeutic target for improving ablation outcomes in AF [29–33]. Therefore, correctly detecting a clinical focal impulse or localized rotors via phase mapping would be a complementary step to targeting and ablating the AF drivers [34]. In our study, the location-centric method produced a similar trajectory as that of the Iyer-Gray method. The highest HD value among all of our results was 3.9 mm, while the typical lesion size of radiofrequency ablation is approximately 10 mm [35]. Therefore, our method is feasible for identifying PS sites in translational applications.

### Automated PS calculation methods and unmet needs

Developing an automated detection algorithm is important for clinical applications [29, 30]. In clinical applications, computational efficiency becomes important. Conventional methods for PS calculation that are based on the Iyer-Gray method are quite inefficient computationally. Recently, several research groups have developed algorithms for PS detection in clinical and experimental studies by unipolar or bipolar recordings. Zou et al. [15] developed a PS tracking method that combines automated image analysis and mathematical convolutions, based on the method of Bray et al. [13]. This multi-step approach that includes manual verification has improved the accuracy of PS detection. Umapathy et al. [10] reviewed the calculation and use of phase mapping from unipolar electrograms for detecting PS. Kuklik et al. applied a Hilbert transform to construct a phase with unipolar electrograms [36]. Our approach adopted a simplified and insightful method by identifying the point that satisfies the discontinuity condition. It is interesting to note that such a simple approach successfully identified PS points, with minimal difference compared to the conventional method.

One great advantage of this new method is that it does not require voltage information of neighboring sites, which is necessary for the current Iyer-Gray method. Furthermore, there is no standard way to formulate line integrals in 3D meshes with irregular grid point spacing, which is necessary for organ-scale finite element simulations with realistic atrial and/or ventricular geometry.

The Iyer-Gray algorithm needs information of neighboring sites, which cannot be always adjacent in the memory space. Thus, the memory overhead is unavoidable in the implementation of the Iyer-Gray method. In contrast, our algorithm does not require neighboring information. The reduced memory overhead is a major cause of the dramatic speed-up of our algorithm. Additionally, independency from adjacent nodes enables much more efficient parallel implementation on a multi-core CPU or a general-purpose GPU (GPGPU). Since, inter-node memory interference is unnecessary, our algorithm can be easily optimized for parallel computing by avoiding parallel processing overhead.

### Future directions

Our results are promising, as our proposed method can be applied to realistic three-dimensional (3D) heart models with unstructured or irregular grid. Furthermore, as our approach has potential to reduce computation time for PS calculation, it is feasible for application in PS calculation with a realistic 3D structure model consisting of huge amounts of data points (millions of nodes) [37, 38]. The availability of faster tools for calculating PS may dramatically contribute to the translational applications of virtual PS mapping, such as PS-guided ablation [32, 39, 40].

Mathematically, our new method detects temporal singular points of the phase function θ(x, y, t), while the original concept of the phase singular point is spatial singularity. Since the phase function θ(x, y, t) is continuous almost everywhere in the spatiotemporal space, temporal singularity may be closely related to spatial singularity. This remains to be proved mathematically.

### Limitations

Our method assumes that voltage changes continuously over time. Therefore, it has a conceptional limitation in that the core is in the static and non-excited state over time, and cannot localize PS for a site without changing in phase over time. However, the static and non-excited core of the spiral wave is different from PS site at which wavefront and wavetail meet [7]. Thus, our method is still applicable to detect PS sites of functional reentries.

We calculated V_mean_ value as the average value of the action potential among the entire time records. This method for calculating V_mean_ is a critical barrier to simultaneous analysis of fibrillation simulation and PS detection. The selection of origin value was profoundly investigated by Gray et al. [41] using the FitzHugh-Nagumo model. However, pre-selection of V_mean_ on the realistic cardiac myocyte model has been poorly studied. For further investigation, we propose utilizing either the empirical value or the average of single action potential for pre-selecting V_mean_.

We have tested our proposed method by simulating spiral waves in a 2D model of atrial tissue, which assumes a homogeneous medium. Inhomogeneity due to the anatomical structure, fiber orientation, or fibrotic lesions [42] may lead to discontinuity in the activation of action potential. Therefore, performance of the proposed location-centric method for detection of PS points should be tested in such more realistic scenarios of cardiac fibrillation. Thus, the method may need to be adjusted for constructing phase maps, an issue that we have not explored yet.

## Conclusion

We have developed a novel method for detection and calculation of PS points, which is more efficient than the conventional Iyer-Gray method. The proposed algorithm for location-centric calculation of PS points is accurate and robust with respect to the scenarios of control and electrical remodeling.

## Supporting Information

S1 Appendix Pseudocode. (PDF)

S1 Fig Additional snapshots of phase singularity detection. We provided two snapshots of voltage map, membrane potentials, phases and phase differences for control and 0.3×I_CaL_. In these examples, the two methods show different PS points (red: Iyer-Gray method, black: location-centric method). The red dotted plot was recorded from the closest PS point, calculated by the Iyer-Gray method, to the other PS point that was calculated by the location-centric method.
(TIF)

S2 Fig Effects of sampling interval on PS calculation between the two methods. We compared PS maps between the Iyer-Gray method and the location-centric method for various sampling time interval (T = 0.1 ms, 0.5 ms, 1 ms, 5 ms, and 10 ms).
(TIF)
