# Supplementary material for: A New Efficient Method for Detecting Phase Singularity in Cardiac Fibrillation
Source: PLoS One. 2016 Dec 1;11(12):e0167567. doi: 10.1371/journal.pone.0167567 (PMC5131933; doi:10.1371/journal.pone.0167567)
Supplement: S1 Appendix — (PDF) [file pone.0167567.s001.pdf]

S1 Appendix: Pseudocode

We provide pseudocodes of the location-centric method and the Iyer-Gray method for detecting phase singularity on the N×N 2D grid.

Location-centric method

```
for i = 1 to N
    for j = 1 to N
        V_mean ← Average(V(i, j, 1), V(i, j, 2), …, V(i, j, T))

        for t = 1 to T
            θ(t) ← Atan2(V(i, j, t+τ)-V_mean, V(i, j, t)-V_mean)

        for t = 1 to T-1
            if θ(t+1) - θ(t) < M
                PhaseSingularity(i, j, t) ← True
            else
                PhaseSingularity(i, j, t) ← False
```

Iyer-Gray method

```
PhaseDifference(a, b):
if |a-b| ≤ π
    return a-b
else if a-b > 0
    return a-b-2π
else
    return a-b+2π


PhaseSingularity-Detection:
for i = 1 to N
    for j = 1 to N
        V_mean ← Average(V(i, j, 1), V(i, j, 2), …, V(i, j, T))
        for t = 1 to T
            θ(i, j, t) ← Atan2(V(i, j, t+τ)-V_mean, V(i, j, t)-V_mean)

for i = 2 to N-1
    for j = 2 to N-1
        for t = 1 to T
            K11 ← PhaseDifference(θ(i-1, j, t), θ(i-1, j-1, t))
            K21 ← PhaseDifference(θ(i-1, j+1, t), θ(i-1, j, t))
            K31 ← PhaseDifference(θ(i, j+1, t), θ(i-1, j+1, t))
            K32 ← PhaseDifference(θ(i+1, j+1, t), θ(i, j+1, t))
            K33 ← PhaseDifference(θ(i+1, j, t), θ(i+1, j+1, t))
            K23 ← PhaseDifference(θ(i+1, j-1, t), θ(i+1, j, t))
            K13 ← PhaseDifference(θ(i, j-1, t), θ(i+1, j-1, t))
            K12 ← PhaseDifference(θ(i-1, j-1, t), θ(i, j-1, t))
            K ← K11 + K21 + K31 + K32 + K33 + K23 + K13 + K12
            if K = 2π or -2π
                PhaseSingularity(i, j, t) ← True
            else
                PhaseSingularity(i, j, t) ← False
```
