# Supplementary figures and images for: A New Efficient Method for Detecting Phase Singularity in Cardiac Fibrillation
Source: PLoS One. 2016 Dec 1;11(12):e0167567. doi: 10.1371/journal.pone.0167567 (PMC5131933; doi:10.1371/journal.pone.0167567)

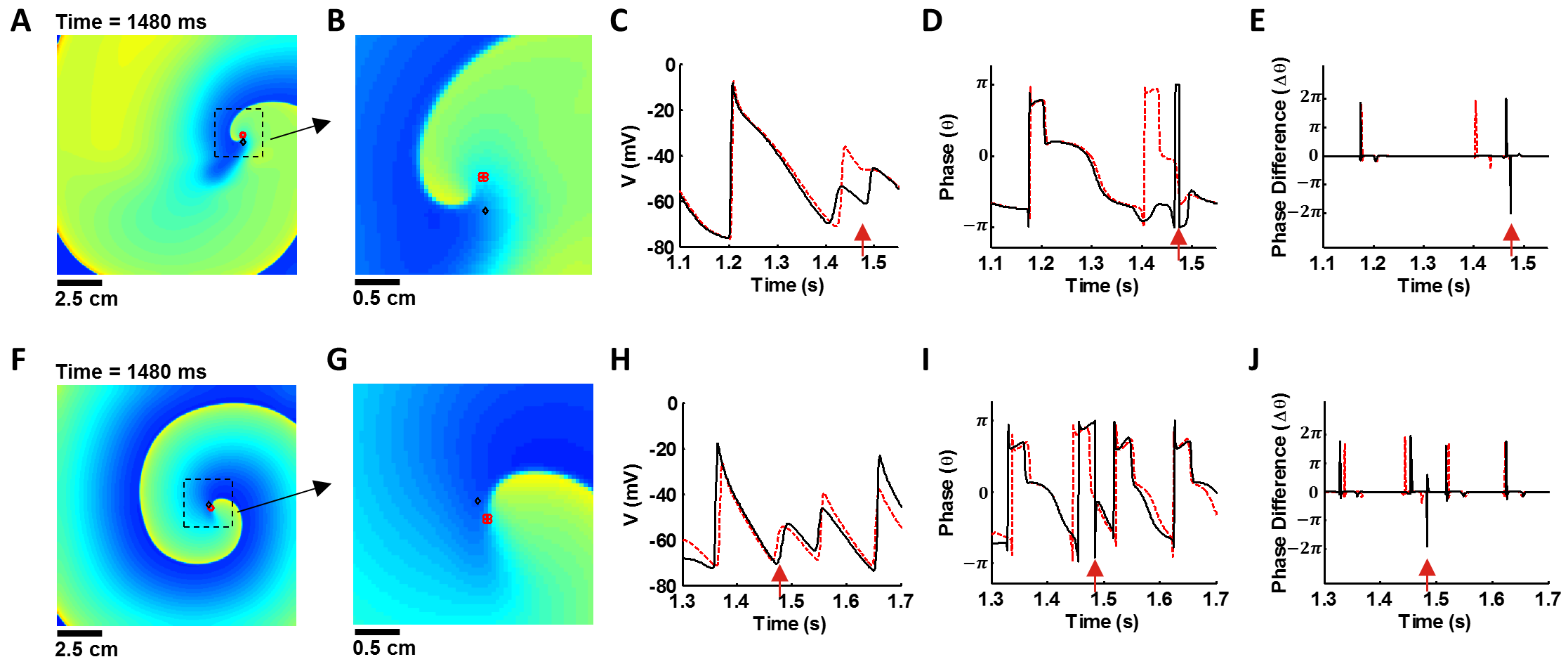

Supplement: S1 Fig — We provided two snapshots of voltage map, membrane potentials, phases and phase differences for control and 0.3×ICaL. In these examples, the two methods show different PS points (red: Iyer-Gray method, black: location-centric method). The red dotted plot was recorded from the closest PS point, calculated by the Iyer-Gray method, to the other PS point that was calculated by the location-centric method. (TIF) [file pone.0167567.s002.tif]

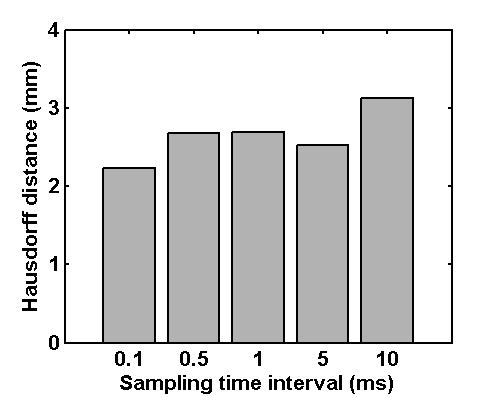

Supplement: S2 Fig — We compared PS maps between the Iyer-Gray method and the location-centric method for various sampling time interval (T = 0.1 ms, 0.5 ms, 1 ms, 5 ms, and 10 ms). (TIF) [file pone.0167567.s003.tif]
